# Sociocultural Factors Influencing the Management of Meningitis Among Older Adults in Kwara State, Nigeria

**DOI:** 10.1093/geroni/igab046.907

**Published:** 2021-12-17

**Authors:** Kafayat Mahmoud, Moshood Issah, Hameed Bakare

**Affiliations:** 1 University of Kansas, Lawrence, Kansas, United States; 2 University of Ilorin, Ilorin, Kwara, Nigeria; 3 University of Ilorin, University of Ilorin, Kwara, Nigeria

## Abstract

During epidemic and non-epidemic seasons, the Kwara North, Nigeria, has consistently reported high incidence rates for meningitis, a disease which mostly affects older members of the community. Limited studies have investigated the nexus between climate change-meningitis and socio-cultural factors influencing the management and control of meningitis among the older adults. This study explored the lived experiences of older individuals with meningitis and relationships with their caregivers in Kaiama Local Government area of Kwara state, Nigeria. 15 participants, 6 men and 9 women, aged 65+ years were purposively selected, and in-depth interviews were conducted. Results indicated that most of the older adults believed that the disease is caused by spiritual or supernatural forces (such as witchcrafts, demons, evil spirits among others), and the treatment and management, using orthodox medicine of it has been hampered by certain socio-cultural beliefs. Due to beliefs about contagion, older adults are mostly left on their own, with adult children occasionally visiting their parents to provide care only to return to their own homes. Also, children visit with traditional healers who perform rites of purification and give older adults concoctions to use. The study concluded that meningitis is one of the leading causes of untimely death of some older adults in the study communities and it has been worsened by some socio-cultural practices. Based on this conclusion, the study recommended massive enlightenment of the general public about best practices in the treatment and management of the disease among the older members of the study communities.

